# Predictive Factors of Sensitivity to Elisidepsin, a Novel Kahalalide F-Derived Marine Compound

**DOI:** 10.3390/md11030944

**Published:** 2013-03-20

**Authors:** Maria Serova, Armand de Gramont, Ivan Bieche, Maria Eugenia Riveiro, Carlos Maria Galmarini, Miguel Aracil, José Jimeno, Sandrine Faivre, Eric Raymond

**Affiliations:** 1 AAREC Filia Research, 1, Paul Verlaine, Boulogne Billancourt 92100, France; E-Mails: mserova@aarec-filia-research.com (M.S.); adegramont@aarec-filia-research.com (A.G.); 2 INSERM U728 and Departments of Medical Oncology, Beaujon University Hospital (AP-HP-Paris 7 Diderot), 100, bd General Leclerc, Clichy 92110, France; E-Mails: eugenia.riveiro@oncotd.com (M.E.R.); sandrine.faivre@bjn.aphp.fr (S.F.); 3 Laboratory of Molecular Genetics, Beaujon University Hospital, Paris 7 Diderot, 100, bd General Leclerc, Clichy 92110, France; E-Mail: ivan.bieche@parisdescartes.fr; 4 Cell Biology Department, PharmaMar, Avda de los Reyes 1, Pol. Ind. La Mina, Colmenar Viejo (Madrid) 28770, Spain; E-Mails: cgalmarini@pharmamar.com (C.M.G.); maracil@pharmamar.com (M.A.); jjimeno@pangaeabiotech.com (J.J.)

**Keywords:** Irvalec, oncosis, E-cadherin, ErbB3

## Abstract

Elisidepsin (PM02734, Irvalec^®^) is a synthetic marine-derived cyclic peptide of the Kahalalide F family currently in phase II clinical development. Elisidepsin was shown to induce rapid oncosis in ErbB3-expressing cells. Other predictive factors of elisidepsin sensitivity remained unknown. A panel of 23 cancer cell lines of different origin was assessed for elisidepsin cytotoxicity and correlated with mutational state, mRNA and protein expression of selected genes. Elisidepsin showed potent and broad cytotoxic effects in our cancer cell line panel, being active at concentrations ranging from 0.4 to 2 μM that may be relevant for clinical settings. We have shown that elisidepsin is more active in cells harboring epithelial phenotype with high E-cadherin and low vimentin expression. In addition, high ErbB3 and Muc1 expression was correlated with sensitivity to elisidepsin, whereas the presence of KRAS activating mutations was associated with resistance. In DU-PM cells with acquired resistance to elisidepsin, ErbB3 expression was decreased, while Bcl2 was increased. DU-PM cells displayed higher sensitivity to ErbB1-inhibitors suggesting possible cross-talk of ErbB1 and ErbB3 signaling pathways. Combinations of elisidepsin with lapatinib and several chemotherapies including 5-FU and oxaliplatin resulted in synergistic effects that offer the potential of clinical use of elisidepsin in combination settings.

## 1. Introduction

Natural products have been a common source of anticancer drugs. Kahalalide F (KF) belongs to a family of natural depsipeptides that were first isolated from the Hawaiian herbivorous marine sacoglossan mollusk *Elysia rufescens* (Plakobranchidae) and later from its green algal diet of *Bryopsis pennata* (Bryopsidaceae) [1]. KF is the largest and the most biologically active cyclic peptide of the 13 natural peptides isolated from *E. rufescens*. Treatment of cancer cells with KF resulted in dramatic changes in lysosomal membranes and large vacuoles, leading to cell swelling [2,3]. Preclinical studies have shown strong activity of KF against cell lines and tumor specimens derived from different human solid tumors, including non-small cell lung cancer (NSCLC), prostate, breast, ovarian, and colon carcinomas. Activity of KF was correlated with ErbB3 (HER3) expression and was associated with the inhibition of the downstream phosphatidylinositol 3-kinase (PI3K)-Akt signaling pathway [4,5] in sensitive cell lines. Ectopic expression of a constitutively active Akt mutant reduced KF cytotoxicity [5].

Elisidepsin (PM02734, Irvalec^®^) is a novel synthetic agent derived from KF that is currently being evaluated in phase I/II clinical trials. As for KF, recent studies have shown a correlation between elisidepsin sensitivity and the expression of the ErbB3 receptor in a panel of NSCLC and other cell lines [6,7]. Elisidepsin exposure was shown to induce downregulation of ErbB3 protein expression in most cell lines. Furthermore, elisidepsin was more effective in the induction of ErbB3 dephosphorylation and degradation than that of ErbB2 (HER2) and ErbB1 (EGFR) in human NSCLC cell lines. Nonetheless, a recent paper contested this result by showing that overexpression of ErbB2 and ErbB3 in CHO cells, or knock-down of ErbB3 expression by RNA interference, did not alter elisidepsin sensitivity [8], thereby suggesting that other potential targets could mediate elisidepsin sensitivity. *In vitro*, it was recently reported that elisidepsin induces a rapid loss of membrane integrity in tumor cells, accompanied by a significant Ca^2+^ influx, perturbations of membrane conductivity, severe swelling, and the formation of giant membranous vesicles [9]. Using the yeast *S. cerevisiae* as a model to investigate elisidepsin mode of action, Herrero and colleagues [10] found that many genes involved in intracellular trafficking, mitochondrial functions, cell-wall and sphingolipid biosynthesis were involved in elisidepsin sensitivity. In human cell lines, they showed that overexpression of the fatty acid 2-hydroxylase (FA2H) increased elisidepsin sensitivity, whereas its knock-down increased cell resistance [10]. The above results, associated with the observation by Varadi and colleagues that elisidepsin induced characteristic changes in the organization of the plasma membrane, suggested to the authors that the observed modification in the ErbB receptors signaling pathways were only the consequences of the initial cell membrane alterations [8]. Nonetheless, they showed that some changes were specific to ErbB3, such as the increased sensitivity to an ErbB3 conformation-sensitive antibody or the specific internalization of the protein. Whether these specific changes are directly or indirectly linked to elisidepsin activity will need further investigations.

Pharmacokinetic analysis showed that safe plasma concentrations up to 2 μM could be achieved in patients with advanced cancers [11,12]. The finding of one complete response and cases of long-lasting stable disease in phase I trials has prompted a phase Ib/II trial in patients with advanced/metastatic non-small cell lung, esophageal and gastric cancers. Molecular determinants of elisidepsin sensitivity and resistance remain to be elucidated in order to provide clinical orientations identifying tumor types that may benefit from elisidepsin therapy.

In this study, we characterized the cytotoxicity of elisidepsin in a panel of human cancer cell lines and, to improve its potential use in clinic, we described potential predictive molecular markers of sensitivity and resistance.

## 2. Results

### 2.1. Antiproliferative Effects of Elisidepsin

Cytotoxic effects of elisidepsin were evaluated in a panel of 23 cancer cell lines of different origin after 72 h of drug exposure (Table 1).

**Table 1:** IC50s of elisidepsin in a panel of human cancer cell lines.

Cell line	Tumor type	IC50 (μM)
ZR-75-1	Breast	0.40 ± 0.1
SKBR3	Breast	0.50 ± 0.1
MDA-MB-361	Breast	1.25 ± 0.3
MDA-MB-231	Breast	4.70 ± 1.2
MCF7	Breast	8.00 ± 2.7
Colo205	Colon	0.75 ± 0.2
HCC2998	Colon	1.20 ± 0.4
HT29	Colon	3.70 ± 0.8
Colo205R	Colon	6.10 ± 2.1
HCT116	Colon	7.20 ± 2.2
SQ20B	Head and Neck	3.50 ± 1.1
HEP2	Head and Neck	4.30 ± 1.2
SCC61	Head and Neck	5.60 ± 1.8
SK-HEP1	Hepatocarcinoma	6.00 ± 1.9
HOP62	Lung	6.30 ± 1.9
HOP92	Lung	8.00 ± 2.9
MDA-MB-435	Melanoma	4.40 ± 0.9
IGROV1	Ovarian	4.20 ± 0.8
OVCAR3	Ovarian	7.30 ± 2.2
CAPAN1	Pancreas	5.00 ± 1.6
MiaPaCa2	Pancreas	8.80 ± 3.1
DU145	Prostate	1.26 ± 0.4
PC3	Prostate	1.80 ± 0.4

**Figure 1:**
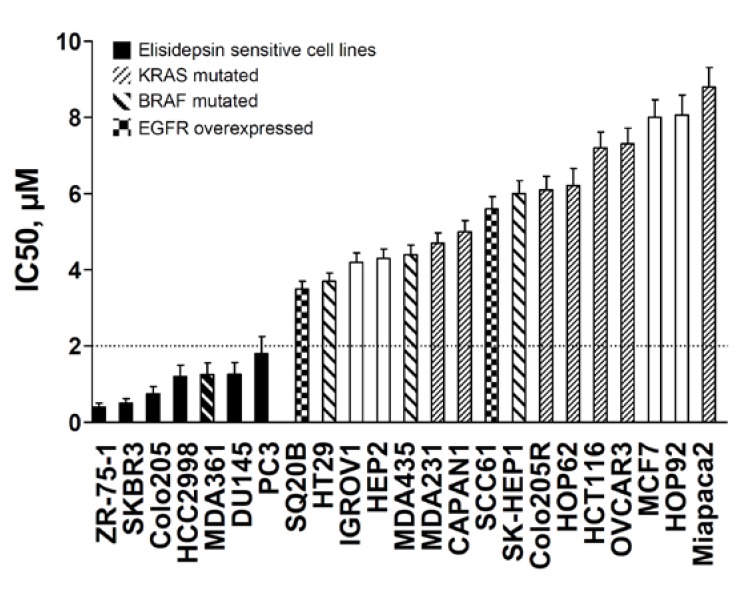
Antiproliferative effects of elisidepsin in a panel of human cancer cell lines with their associated mutations in KRAS and BRAF genes and EGFR overexpression status. IC50s of elisidepsin were determined using MTT assay in cell lines exposed for the drug concentrations for 72 h. Black bars represent sensitive cell lines 0.4 μM < IC50s < 2.0 μM. KRAS and BRAF mutations, as well as EGFR overexpression, are displayed by specific patterned bars.

From phase I pharmacokinetic studies, elisidepsin has been proven to be active and safely administered up to 2 μM of plasma concentration. Based on these clinical data, the cell lines were separated into two groups (Figure 1): The high sensitive cell lines with IC50s below 2 μM, ranging from 0.4 μM to 2 μM, and the low sensitive cell lines, with IC50s over 2 μM, ranging from 3.5 μM to 8.8 μM. IC50 values obtained at different time intervals (24 h and 48 h) were similar indicating that elisidepsin exerted its cytotoxic effects immediately after drug exposure (Supplementary Table S1).

To examine the effects of elisidepsin on cellular proliferation, we performed a cell cycle analysis in the highly sensitive SKBR3 breast cancer cell line (IC50 = 0.5 μM) treated with increasing concentrations of elisidepsin from 0.125 μM to 1 μM (Supplementary Figure S1). At all concentrations, the proportion of cells in all the cell cycle phases were decreased due to the increasing amount of dying cells represented by the sub-G1 cell population. The proportion of dying cells was dose dependent, reaching 80% at 1 μM elisidepsin. Similar results were obtained with the DU145 cell line (data not shown).

These observations prompted us to examine if cell death induced by elisidepsin was the result of apoptosis. In the sensitive DU145 prostate cancer cell line, we first tested for an increase in annexin V staining upon elisidepsin exposure. As shown in Supplementary Figure S2, the annexin V staining increased approximately threefold upon treatment with 1 μM elisidepsin for 24 or 48 h. We then tested for activation of the apoptotic proteins caspase-9 and PARP by looking at their cleaved products. As positive control, we treated DU145 cells with increasing methotrexate concentrations which are known to induce apoptosis. No cleaved caspase-9 and PARP were detected after 24 h elisidepsin treatment (Supplementary Figure S2) suggesting that elisidepsin-induced cell death may not be the result of the apoptotic machinery activation. Oncosis is another pathway of cell death characterized by cell swelling and loss of cell membrane integrity, which can also account for the increase of annexin V staining [13]. When treated with elisidepsin, DU145 cells were clearly subjected to cell swelling with the apparition of large vesicles extruding from the plasma membrane (Supplementary Figure S3). To confirm that elisidepsin exposure was associated with a loss of cell membrane integrity, propidium iodide was added to the culture medium and its concentration within the cells was measured by fluorimetry. Rapid PI uptake occurred at elisidepsin concentrations of 0.5 μM and above, suggesting that elisidepsin affected the permeability of these tumor cells in a time- and concentration-dependent manner (Supplementary Figure S3). These results may suggest that elisidepsin induced oncosis type of cell death instead of apoptosis.

### 2.2. ErbB3*,* E-cadherin*,* Muc1 and the MAPK Activation Predict Elisidepsin Sensitivity and Resistance

**Figure 2:**
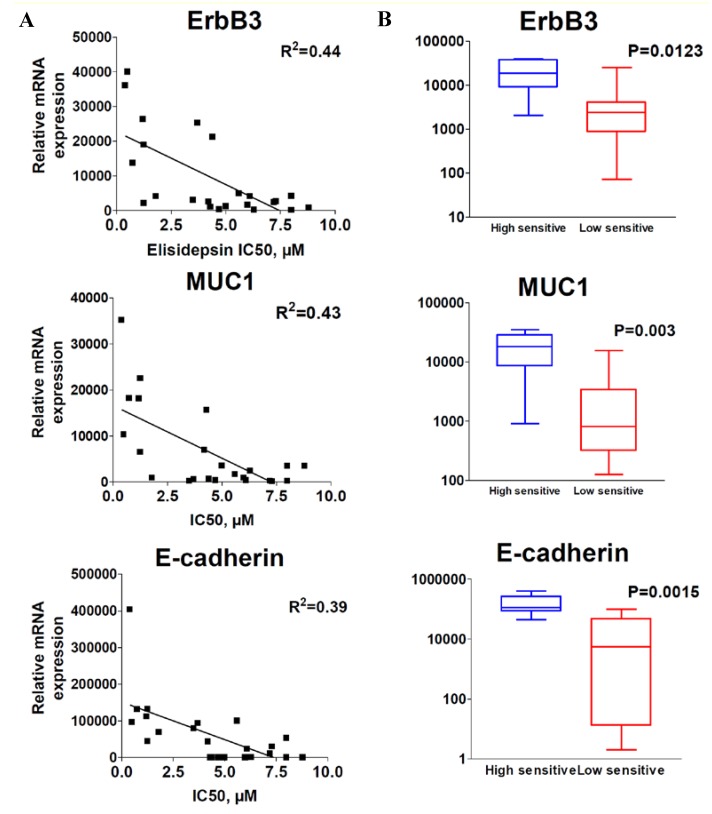
Correlation of ErbB3, Muc1 and E-cadherin with elisidepsin sensitivity. Correlation of mRNA expression (qRT-PCR) with elisidepsin IC50s (**A**) and differential expression in high sensitive and low sensitive groups of cell lines (**B**).

To identify potential genes whose expression may correlate with elisidepsin sensitivity, a panel of more than 70 genes (Supplementary Table S2) was analyzed by qRT-PCR and compared with elisidepsin sensitivity in the above panel of 23 cancer cell lines. Data analysis indicated that a high level of ErbB3, Muc1 and E-cadherin mRNA expression correlated with elisidepsin sensitivity (Figure 2A, *R*^2^ = 0.44, 0.43 and 0.39, respectively).

Since ErbB3 must heterodimerize with the other members of the ErbB network for its activity, we compared the relative mRNA expression levels of ErbB1, ErbB2, ErbB3 and ErbB4 in elisidepsin high sensitive and low sensitive cells (Supplementary Figure S4 and Supplementary Table S3). We also analyzed the mutated status of KRAS and BRAF genes (Figure 1).

Two cell lines seemed to overexpress ErbB1, SQ20B and SCC61, but no correlation was found between the expression level of ErbB1 and the sensitivity to elisidepsin (*R*^2^ < 10^−3^). Despite this lack of correlation, we noticed that the sensitive cells were less subjected to MAPK pathway constitutive activation through either KRAS and BRAF mutations or ErbB1 overexpression (Figure 1). In fact, only one cell line among the sensitive cell lines displayed a BRAF mutation (1/7 = 14%), whereas 75% (12/16) of the other cell lines displayed mutation in KRAS and BRAF genes or overexpression of ErbB1 (*p* = 5 × 10^–3^).

ErbB2 protein overexpression in association with ErbB2 gene amplification is an excellent biomarker for anti-ErbB2 therapies in breast and gastric cancers [14,15]. In our panel of cancer cell lines, there were four breast cancer cell lines (ZR-75-1, SKBR3, MDA-MB-231, and MCF7) among which only two cell lines were considered to overexpress ErbB2, SKBR3, which is scored at 3+, and ZR-75-1, which is scored at 2+. These two cell lines were also the only ones that overexpressed ErbB2 in our gene expression analysis (Supplementary Table S3). Besides these two cell lines, no correlation was found between ErbB2 gene expression and elisidepsin sensitivity. ErbB4 was poorly expressed in most cell lines, except SKBR3 and ZR-75-1, but no clear correlation was found with elisidepsin sensitivity.

### 2.3. Elisidepsin Sensitivity Is Dependent on Epithelial-to-Mesenchymal Transition (EMT)

**Figure 3:**
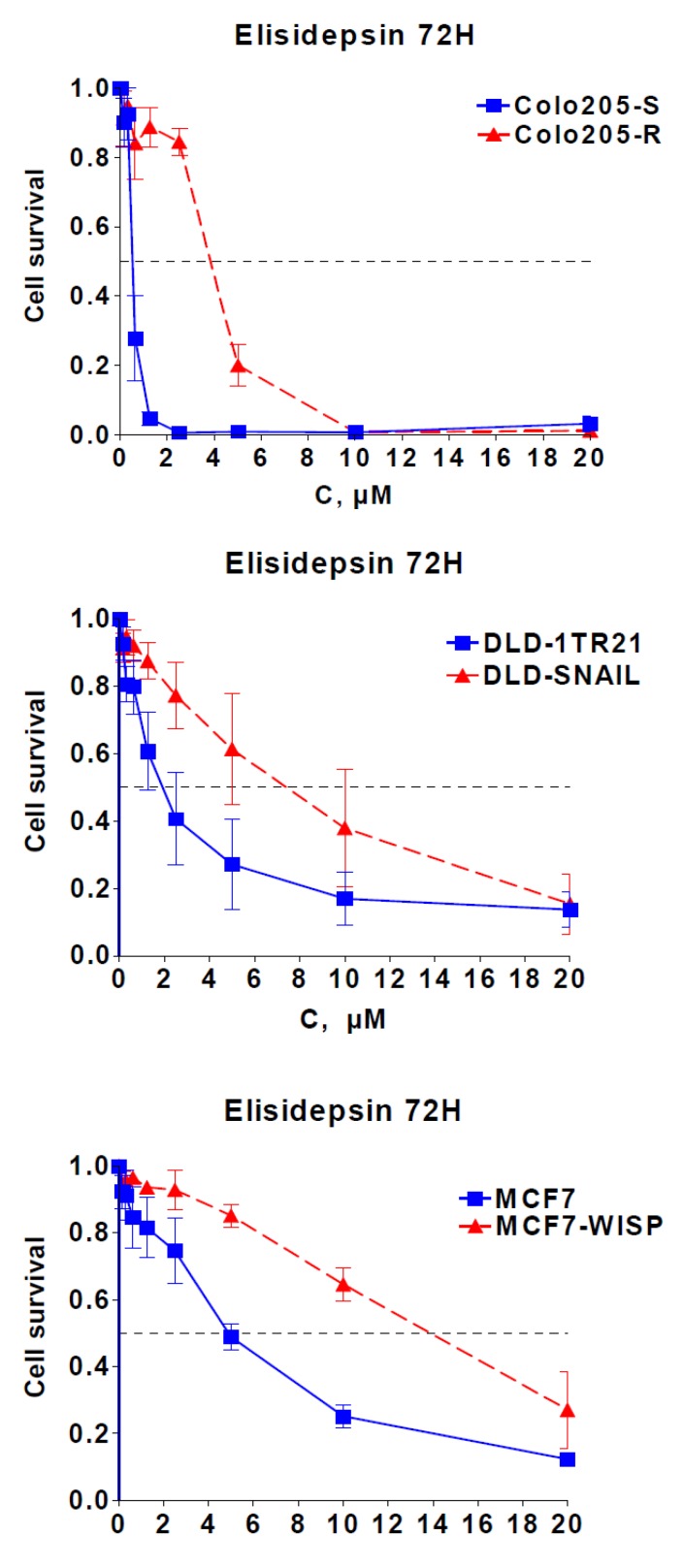
Role of EMT in elisidepsin activity. Cytotoxicity of 72 h elisidepsin in three isogenic EMT models: Colo205-S/Colo205-R; DLD-1TR21/DLD-Snail; MCF7/MCF7-Wisp.

Correlation of elisidepsin sensitivity with E-cadherin, Muc1 and ErbB3 suggested a role of the epithelial/mesenchymal cell differentiation in cellular response. This was reinforced by the fact that in our panel, the mesenchymal Colo205-R cell line, established from the epithelial cell line Colo205-S [16,17], was eight times more resistant to elisidepsin than its parental counterpart (Figure 1, Figure 3).

To gain insight into the role of epithelial/mesenchymal cell differentiation in elisidepsin antiproliferative activity, we studied elisidepsin effects in two different models inducible for epithelial-to-mesenchymal transition (EMT): DLD-1TR21-hSnail, which contain an inducible SNAIL gene [18], and MCF7-shWISP knock-down for the WISP-2 gene [19]. SNAIL induction in the DLD1 colorectal cancer cells and knock-down of WISP-2 by RNA interference in MCF7 breast cancer cells (Supplementary Figure S5) led to morphologic changes [18,19] that coincide with induction of EMT and resistance to elisidepsin (Figure 3); inactivation of WISP or induction of SNAIL led to a 3–4 fold decrease in elisidepsin sensitivity in both models (1.7 μM *vs*. 9.0 μM in DLD/DLD-Snail and 5.3 μM *vs*. 18 μM in MCF7/MCF7-Wisp). These data are in accordance with high E-cadherin being a predictive factor of elisidepsin sensitivity and suggest that elisidepsin may primarily target epithelial cells.

### 2.4. Characterization of Acquired Resistance to Elisidepsin

**Figure 4:**
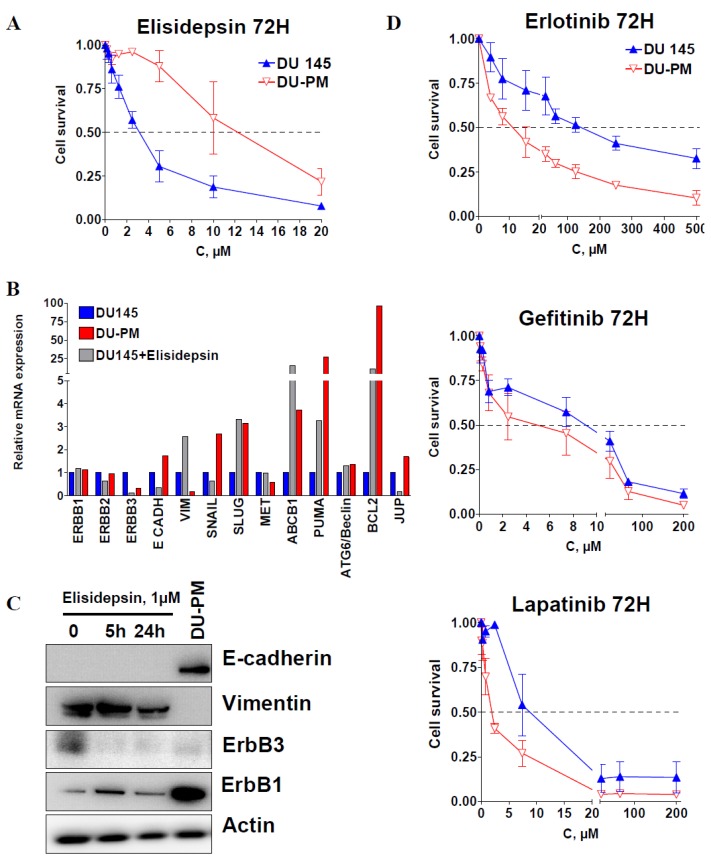
Resistance to elisidepsin sensitizes cells to tyrosine kinase inhibitors. Cytotoxicity of 72 h elisidepsin in DU145 and its resistant counterpart (DU-PM) (**A**). Differential relative mRNA expression (qRT-PCR) of several genes in DU145, DU-PM cells and DU145 exposed to 1 μM elisidepsin for 24 h (DU145 + elisidepsin) (**B**). Protein expression levels of E-cadherin, vimentin, ErbB3 and ErbB1 in DU145 cells exposed to 1 μM elisidepsin for 5 and 24 h, and in DU-PM cells (**C**). Cytotoxicity of 72 h ErbB1 inhibitors erlotinib, gefitinib and ErbB1/2 inhibitor lapatinib in DU145 and DU-PM cells (**D**).

To further investigate the mechanisms of elisidepsin resistance, we developed the DU145 cell line with acquired resistance to the drug. DU145 cells were exposed to stepwise increasing concentrations of elisidepsin for six months. The resulting cell line, DU-PM, displayed an IC50 of 13 μM, *i.e.*, was over four times more resistant to elisidepsin than its parental cell line (Figure 4A).

After five passages in drug-free medium, DU-PM cells retained drug resistance. We then compared the mRNA expression levels of several genes involved in cell adhesion, signal transduction, proliferation and cell survival between DU145 cells, DU145 cells treated with 1 μM elisidepsin for 24 h and the elisidepsin-resistant cells DU-PM (Figure 4B). In DU145 cells treated with 1 μM elisidepsin for 24 h, gene and protein expression of both ErbB3 and E-cadherin were clearly reduced (Figure 4B), EGFR and ErbB2 gene expression stayed unchanged, whereas increased gene expression was observed for the mesenchymal markers Vimentin and Slug, the apoptosis regulators Puma and Bcl2, and the ABC transporter ABCB1 involved in multidrug resistance. Interestingly, short-term treatment with elisidepsin resulted in an increased expression of p21 that was retained in long-term treatment (data not shown). In contrast to cells treated with elisidepsin, in cells resistant to elisidepsin, E-cadherin mRNA and protein expression increased (Figure 4B,C), whereas the expression of vimentin was diminished. Since DU-PM cell resistance to elisidepsin seemed to be associated with a more epithelial phenotype (low vimentin and high E-cadherin), we assessed the protein expression of four other EMT markers to confirm our observation (Supplementary Figure S6). Mesenchymal transcription factors Slug and ZEB1 were differentially expressed in “response” to elisidepsin. Whereas Slug was increased in DU-PM cells, ZEB1 was decreased. Epithelial markers claudin-1 and ZO-1 were both increased in DU-PM compared to parental DU145 cells. DU-PM cells also displayed higher expression of β-catenin and ErbB1 (Figure 4C, Supplementary Figure S6), whereas protein expression levels of Snail, ErbB2 and ErbB4 were not detectable. These results showed that DU-PM cells exhibited a phenotype that was clearly more epithelial than the parental counterpart, suggesting that other factors than the mesenchymal signature of the cells may be involved in elisidepsin resistance such as cell death inhibition and ErbB1 expression.

These observations prompted us to test whether or not an increase in the E-cadherin/vimentin ratio and ErbB1 expression in DU-PM cells compared to DU145 parental cells will sensitize cells to ErbB1 inhibitor erlotinib. We thus treated DU145 and DU-PM cells with increasing concentrations of erlotinib, gefitinib or lapatinib. Figure 4D indeed shows that long-term treatment of DU145 with elisidepsin increased its sensitivity to the EGFR TKI inhibitors.

### 2.5. Elisidepsin Combination with Chemotherapies and Targeted Therapies

Considering that colon and prostate cancer cell lines were among the most sensitive cell lines in our panel, the combinations of elisidepsin with 5-FU, oxaliplatin, cisplatin and gemcitabine were evaluated in Colo205 (colon) and DU145 (prostate) cells. The effects of drug combinations on cell proliferation were evaluated using three different schedules: (1) elisidepsin for 24 h followed by chemotherapy for 24 h; (2) chemotherapy for 24 h followed by elisidepsin for 24 h; or, (3) combination of elisidepsin and chemotherapy for 24 h. Combination index (CI) values of <1 indicate synergy, the value of 1 indicates additive effects, and values >1 indicate antagonism (Table 2).

**Table 2:** Combination index (CI) of elisidepsin and 5-FU, gemcitabine, cisplatin, oxaliplatin or lapatinib in three treatment schedules.

Combination schedule		CI, median (min; max)	
		DU145	Colo205
**5-FU-based combinations**	Elisidepsin → 5-FU	0.83 (0.59; 1.14)	1.36 (0.98; 1.52)
	5-FU → Elisidepsin	0.65 (0.49; 0.88)	0.93 (0.71; 1.24)
	Elisidepsin + 5-FU	1.03 (0.59; 1.18)	1.27 (1.14; 1.64)
**Gemcitabine-based combinations**	Elisidepsin → Gemcitabine	1.03 (0.93; 1.15)	-
	Gemcitabine → Elisidepsin	1.01 (0.96; 1.12)	-
	Elisidepsin + Gemcitabine	1.07 (0.90; 1.22)	-
**Cisplatin-based combinations**	Elisidepsin → Cisplatin	1.18 (1.01; 1.38)	-
	Cisplatin → Elisidepsin	1.08 (0.92; 1.36)	-
	Elisidepsin + Cisplatin	1.26 (1.09; 1.38)	-
**Oxaliplatin-based combinations**	Elisidepsin → Oxaliplatin	0.77 (0.75; 0.97)	0.96 (0.92; 3.05)
	Oxaliplatin → Elisidepsin	0.69 (0.33; 0.94)	0.22 (0.08; 1.85)
	Elisidepsin + Oxaliplatin	0.82 (0.69; 0.89)	0.62 (0.19; 1.67)
**Lapatinib-based combinations**	Elisidepsin → Lapatinib	0.98 (0.71; 1.18)	0.98 (0.89; 1.6)
	Lapatinib → Elisidepsin	0.71 (0.34; 1.16)	0.71 (0.67; 0,89)
	Elisidepsin + Lapatinib	1.04 (0.69; 1.12)	0.77 (0.60; 1,21)

The combination of elisidepsin with oxaliplatin in DU145 and Colo205 resulted in synergistic effects (CI < 1) in both cell lines, irrespective of the concentrations used. These effects did not appear to be schedule dependent. The combination of elisidepsin with cisplatin and gemcitabine in DU145 cell line resulted in additive effects for all schedules used with some synergistic effects at high concentrations when elisidepsin was added after chemotherapy (Supplementary Figure S7). Combinations of elisidepsin with 5-FU in DU145 cells were synergistic when elisidepsin was used prior to or after chemotherapy drug and additive for simultaneous exposure. In Colo205 cells, elisidepsin-5FU combinations resulted in additive/antagonistic effects. We also tested the combination of elisidepsin with the tyrosine kinase inhibitor lapatinib, and observed one synergistic combination when elisidepsin followed lapatinib in Colo205 cells. All other combinations displayed additive effects.

## 3. Discussion

In the present study, we used a panel of human cancer cell lines to evaluate elisidepsin’s antiproliferative activity and the molecular determinants of this activity. Elisidepsin showed potent and broad cytotoxic effects in our cancer cell line panel, being active at potential clinically relevant concentrations. Analysis of the effects of elisidepsin cytotoxicity on DU145 and SKBR3 cells showed that cells exposed to elisidepsin lose cell membrane integrity and died from oncosis rather than apoptosis. We show that membrane alteration was very fast since PI uptake, which measures membrane integrity, reaching its plateau in 10 min. It was previously described that HepG2 cells exposed to Kahalalide F displayed important membrane alterations, characterized by cell swelling and rapid permeability to cationic PI [13]. More recently, Ling and colleagues also showed that elisidepsin induced a fast and caspase-independent cell death in non-small-cell lung cancer models [20]. They gave evidence that cell death occurred in part via autophagy involving inhibition of the Akt/mTOR pathway and activation of the death-associated protein kinase (DAPK) [20]. These current evidences suggesting that a different mechanism of cell death may be involved in shedding light on our limited understanding of elisidepsin’s mechanism of action.

However, in parallel to the ongoing mechanistic investigations on elisidepsin mode of action, it is essential for clinical development of the drug to also focus on uncovering potential predictive biomarkers. By screening several dozens of genes in an educated-guess manner, our study revealed some interesting biomarkers of elisidepsin sensitivity and resistance. First, we showed that elisidepsin was more active in cells harboring an epithelial phenotype with high E-cadherin and low vimentin expression. Epithelial-to-mesenchymal transition (EMT) is an essential developmental process by which cells of epithelial origin lose epithelial characteristics and polarity, and acquire a mesenchymal phenotype with increased migratory behavior; these mesenchymal cells have been shown to be more resistant to various chemotherapies and targeted therapies [21,22]. Our findings in inducible EMT models support a role of cell differentiation in drug response, suggesting that EMT may be an important negative predictive factor of sensitivity to elisidepsin. ErbB3 and Muc1, which are proteins mainly expressed in epithelial cells [16], showed a significant positive correlation between mRNA expression and elisidepsin sensitivity supporting our hypothesis that elisidepsin activity depends on cell differentiation. Among the panel of cell lines tested, the combination of high E-cadherin, ErbB3 and Muc1 gene expression predicted the most sensitive cell lines (ZR-75-1, SKBR3, Colo205, HCC2998), whereas high expression of one or two genes among these three were less robust to predict sensitivity to elisidepsin. In fact, regarding the E-cadherin/vimentin ratio, ErbB3 and Muc1 gene expression, MCF7 cells would be predicted to be at least as sensitive as PC3 cells, whereas they are four times less sensitive. Therefore, in addition to the combined overexpression of E-cadherin, ErbB3 and Muc1, other factors may discriminate cell lines in their sensitivity to elisidepsin. Similar results were recently obtained by Teixido and colleagues [23] on the predictive factors of elisidepsin primary resistance. They found a correlation between high ErbB3 expression and epithelial signature of the cells and sensitivity to elisidepsin in different breast and pancreatic cell lines. The authors demonstrated that acquired resistance to elisidepsin in epithelial cells induced phenotypic changes towards a more mesenchymal phenotype. Interestingly, starting with mesenchymal cells, we also observed the changes in cell differentiation with the acquisition of a more epithelial phenotype in DU-PM cells. These results suggest that transitions between epithelial and mesenchymal “states” may be a common mechanism of elisidepsin-acquired resistance. In addition, in HCT116, an elisidepsin-resistant cell line, Teixido and colleagues also observed an increase of ErbB1 expression. Since we showed that with activation of the ErbB1 pathway through ErbB1 overexpression KRAS and BRAF mutations seemed to be associated with primary resistance to elisidepsin, we can hypothesize that ErbB1 overexpression may also be involved in acquired resistance to elisidepsin. In addition to potential predictive markers of sensitivity, we have shown a clear correlation between elisidepsin resistance and KRAS mutational status and, more generally, to EGFR/MAPK pathway activation. In our study, cell lines harboring mutated KRAS were less sensitive to elisidepsin, suggesting that KRAS may be regarded as a negative predictive factor of response to the drug. Interestingly, reviewing previous work by Teixido and colleagues, we found that the author characterized MiaPaCa2, Panc1, HCT116 and HOP62 cell lines as being the most resistant cell lines in their panel [7]. These cells are known to carry activated KRAS mutation, whereas BxPC3, which is a pancreatic cell line with wild type KRAS, was extremely sensitive to the drug. These data support our hypothesis on the role of KRAS in elisidepsin resistance. Considering that KRAS is mutationally activated in approximately 20% of all solid tumors and found associated with resistance to several anticancer agents [24], substantial efforts will have to be made to identify alternative or new drugs counteracting the effects of oncogenic RAS in those subsets of otherwise pharmacologically intractable and clinically refractory human cancers.

Recently, *in vitro* and *in vivo* synergism has been described when combining elisidepsin with a specific EGFR inhibitor erlotinib in NSCLC [6]. In our study, cells with acquired resistance to elisidepsin were more sensitive to erlotinib and lapatinib but not gefitinib than parental cells. The absence of increased sensitivity towards gefitinib may be explained by the fact that DU-PM cells do not carry EGFR mutations (data not shown), which have been linked with gefitinib sensitivity. Combinations of elisidepsin with cisplatin, gemcitabine and docetaxel were shown to be synergistic in a panel of breast, colon and lung cancer cell lines [7].

In our study, oxaliplatin, cisplatin, 5-FU, gemcitabine and lapatinib, administered in combination with elisidepsin, resulted mostly in additive effects with few synergies observed. Sequence-dependent activity was apparent for 5-FU, oxaliplatin and lapatinib, the combination being more active when elisidepsin followed chemotherapy treatment. Considering combination therapy as a critical strategy in the approach to overcoming tumor resistance to therapeutic treatments, elisidepsin used in combination with chemotherapies or ErbB-targeted drugs may improve the effects of cancer treatment.

## 4. Experimental Section

### 4.1. Materials

Elisidepsin was supplied by PharmaMar (Madrid, Spain). Oxaliplatin, cisplatin, 5-FU and lapatinib were purchased from Pharmacy department of Beaujon University Hospital.

### 4.2. Cell Lines

Breast SKBR3, MCF7, MDA-MB-361, MDA-MB-231, ZR-75-1, colon HT29, ovarian OVCAR3, IGROV1, SKOV3, prostate PC3, DU145, hepatocarcinoma SK-HEP1, head and neck SCC61, HEP2, SQ20B, pancreatic MiaPaCa2 and CAPAN1 cell lines were obtained from the ATCC (Rockville, MD, USA). Colon HCT116, Colo205-S, HCC2998, lung HOP62, HOP92, and melanoma MDA-MB-435 cell lines were obtained from the National Cancer Institute collection. Colo205-R cells were developed in our laboratory [16]. DLD-1TR21-hSnail colon cancer cell line contains doxycyclin inducible SNAIL [18], was kindly provided by Geert Berx (Ghent, Belgium). MCF7-shWISP carrying WISP-2 knock-down were kindly provided by Michele Sabbah, Saint-Antoine Hospital (Paris, France) [19]. Cells were grown as monolayers in RPMI medium supplemented with 10% fetal calf serum (Invitrogen, Cergy-Pontoise, France), 2 mM glutamine, 100 units/mL penicillin and 100 μg/mL streptomycin at 37 °C in a humidified 5% CO_2_ atmosphere, and regularly checked for the absence of *Mycoplasma*.

### 4.3. Cell Cytotoxicity Assay

Cell viability was determined using the MTT assay (3-[4,5-dimethylthiazol-2-yl]-2,5-diphenyltetrazolium bromide; Sigma, Saint-Quentin Fallavier, France) [25]. The conversion of yellow water-soluble tetrazolium MTT into purple insoluble formazan is catalyzed by mitochondrial dehydrogenases and is used to estimate the number of viable cells. In brief, cells were seeded in 96-well tissue culture plates at a density of 2 × 10^3^ cells/well. After drug incubation followed by 48-h post-incubation in drug-free medium, cells were incubated with 0.4 mg/mL MTT for 4 h at 37 °C. After incubation, the supernatant was discarded, insoluble formazan precipitates were dissolved in 0.1 mL of DMSO and the absorbance was measured at 560 nm by use of a microplate reader (Thermo, France). Wells with untreated cells or with drug-containing medium only were used as positive and negative controls, respectively. Growth inhibition curves were plotted as the percentage of untreated control cells.

### 4.4. Western Blot Analysis

Cells were lysed in buffer containing 50 mM HEPES (pH 7.6), 150 mM NaCl, 1% Triton X-100, 2 mM sodium vanadate, 100 mM NaF, and 0.4 mg/mL phenylmethylsulfonyl fluoride. Equal amounts of protein (20 μg/lane) were subjected to SDS-PAGE and transferred to nitrocellulose membranes. Membranes were blocked with 5% milk or 5% bovine serum albumin in 0.01% Tween 20/phosphate-buffered saline and then incubated with the primary antibody overnight. Membranes were then washed in TBS-Tween and incubated with the secondary antibody conjugated to horseradish peroxidase. Antibody detection was done with the enhanced chemiluminescence Western blotting detection system. Densitometric analysis was performed under conditions that yielded a linear response. Primary antibodies directed against E-cadherin (24E10), vimentin (D21H3), ErbB3 (1B2E) and actin (8H10D10) were purchased from Cell Signaling (Saint Quentin Yvelines, France) and used at a 1:1000 dilution.

### 4.5. Real-Time RT-PCR

The theoretical and practical aspects of real-time quantitative RT-PCR using the ABI Prism 7900 Sequence Detection System (Perkin-Elmer Applied Biosystems, Foster City, CA, USA) have been described in detail elsewhere [26]. Results were expressed as n-fold differences in target gene expression relative to the TBP gene (an endogenous RNA control) and relative to a calibrator, consisting of the cell line sample from our tested series that contained the smallest amount of target gene mRNA. Experiments were performed in duplicate.

### 4.6. DNA Extraction and Mutation Screening

High-molecular-weight DNA was prepared by standard proteinase K digestion followed by phenol-chloroform extraction. Genomic DNA from the cell lines was amplified with primers specific for KRAS (v-Ki-ras2 Kirsten rat sarcoma viral oncogene homolog; NM_004985) and RAF1 (v-raf-1 murine leukemia viral oncogene homolog 1; NM_002880). PCR was performed with the Taqman PCR Core Reagent Kit. Mutation screening was performed using bidirectional DNA sequencing of purified PCR products with the ABI BigDye terminator sequencing kit on an ABI Prism3130 automatic DNA sequencer. Sequences were aligned with Seqscape^®^ analysis software and were compared with the corresponding reference sequences for genomic DNA (Perkin-Elmer Applied Biosystems).

### 4.7. Determination of Synergistic Activity

Drug combination effects were determined using the Chou and Talalay method [27] based on the median effect principle. Combination index (CI) values of <1 indicate synergy, a CI value of 1 indicates additive effects and a CI value of >1 indicates antagonism. Data were analyzed using the concentration-effect analysis software (Biosoft, Cambridge, UK).

### 4.8. Statistical Analysis

For statistical analysis and graphs, the Instat and Prism software (GraphPad, San Diego, CA, USA) were used. Experiments were performed three times, in duplicate. Means and standard deviations were compared using the Student’s *t*-test (two-sided *p* value).

## 5. Conclusions

Elisidepsin displays an original cytotoxicity profile in a large panel of human cancer cell lines. Epithelial phenotype with high E-cadherin, ErbB3 and Muc1 gene expression may be regarded as predictive factors of sensitivity to elisidepsin, whereas mutated KRAS may induce resistance to this drug. Our data on hypersensitivity of elisidepsin-resistant cells to other ErbB inhibitors offer the potential use of this drug in combination with this class of targeted therapies. Synergy of elisidepsin combinations with several chemotherapies in prostate and colon cancer cells offer the potential of clinical use of this drug in combination settings.

## Supplementary Files

Supplementary File 1 Supplementary Materials (PDF, 908 KB)
