# Supplementary material for: Predictive Factors of Sensitivity to Elisidepsin, a Novel Kahalalide F-Derived Marine Compound
Source: Mar Drugs. 2013 Mar 20;11(3):944–59. doi: 10.3390/md11030944 (PMC3705381; doi:10.3390/md11030944)
Supplement: Supplementary File 1 — Supplementary Materials (PDF, 908 KB) [file marinedrugs-11-00944-s001.pdf]

Supplementary Materials

1. Methods

1.1. Membrane Permeabilization Assays

DU145 cells were cultured at high density in Petri dishes. When confluence was reached, fresh culture medium supplemented with 25 mM HEPES pH 7.4 and 50 μg/mL of propidium iodide (PI), and different concentrations of elisidepsin were added. PI uptake was monitored by fluorescence microscopy. For time-course experiments, the uptake of PI was quantified by plate fluorimetry (531/632 nm) at 37 ℃, up to 70 min (1 min intervals), using a Victor3 Multilabel Counter (Perkin Elmer). Results were expressed as relative fluorescent signals.

1.2. Cell Cycle Analysis

Cell cycle analysis was assessed by flow cytometry. In brief, cells were seeded onto 25 cm^3 flasks and treated with various concentrations of elisidepsin. At various time-points adherent and non-adherent cells were recovered, washed with PBS, fixed in 70% ethanol and stored at 4 ℃ until use. Cells were rehydrated in PBS, incubated for 20 min at room temperature with 250 μg/mL RNAse A, and for 20 min at 4 °C with 50 μg/mL propidium iodide in the dark. The cell cycle distribution and percentage of apoptotic cells were determined with a flow cytometer (FACSCalibur and Cell Quest Pro software BD, Le-Pont-de-Claix, France). The percentages of apoptotic cells were evaluated using the Annexin V-FITC Apoptosis Detection Kit (Sigma, Saint Quentin Fallavier, France).

2. Figures and Tables

Figure S1. Cell cycle distributions of DU145 and SKBR3 cells exposed to 0.125, 0.25, 0.5 and 1 μM elisidepsin, after 48 h treatment.

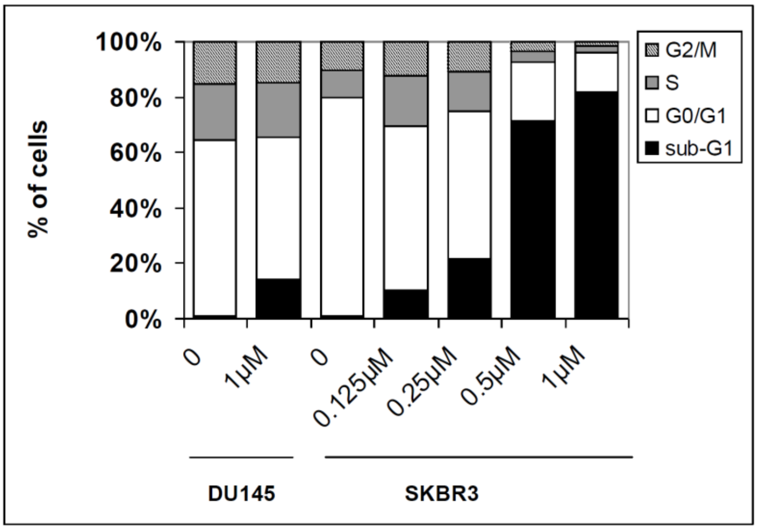

Figure S2. Annexin V staining and PARP and caspase-9 protein expression of DU145 cells after treatment with 1 μM elisidepsin for indicated times.

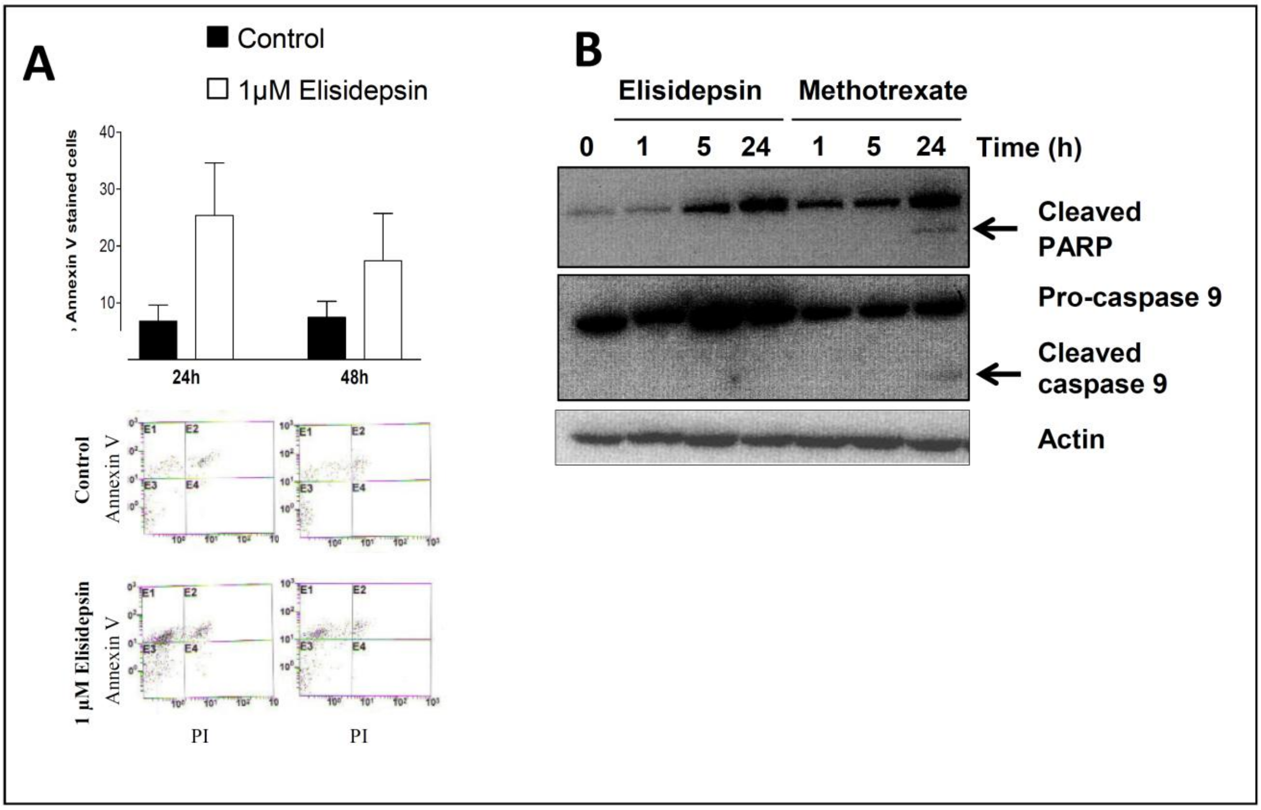

Figure S3. Elisidepsin induction of cell membrane permeability in DU145 cell line. Kinetics of IP incorporation after treatment of cells with 0.1, 0.5, 1 and 5 μM elisidepsin measured by fluorimetry and visualized by fluorescent microscopy. Cell morphology and PI incorporation in DU145 cells treated for 30 min with 5 μM elisidepsin.

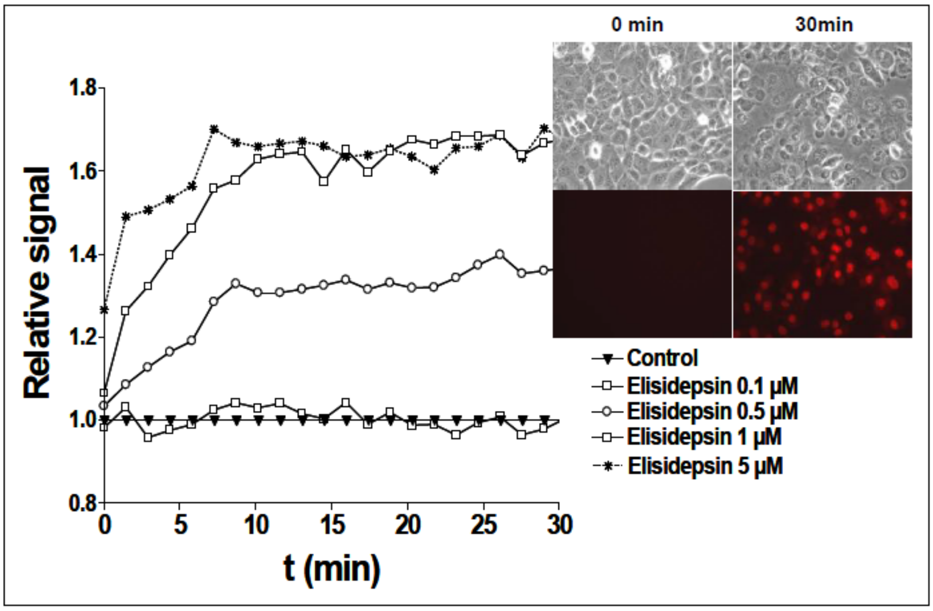

Figure S4. Cell line classification according to their level of gene expression for ErbB1/EGFR, ErbB2, ErbB3, Vimentin, E-cadherin and MUC1. Black circles represent the high sensitive cell lines (**A**). Plot representing correlation of ErbB1 and ErbB3 expression in cell lines with high and low sensitivity to elisidepsin (**B**).

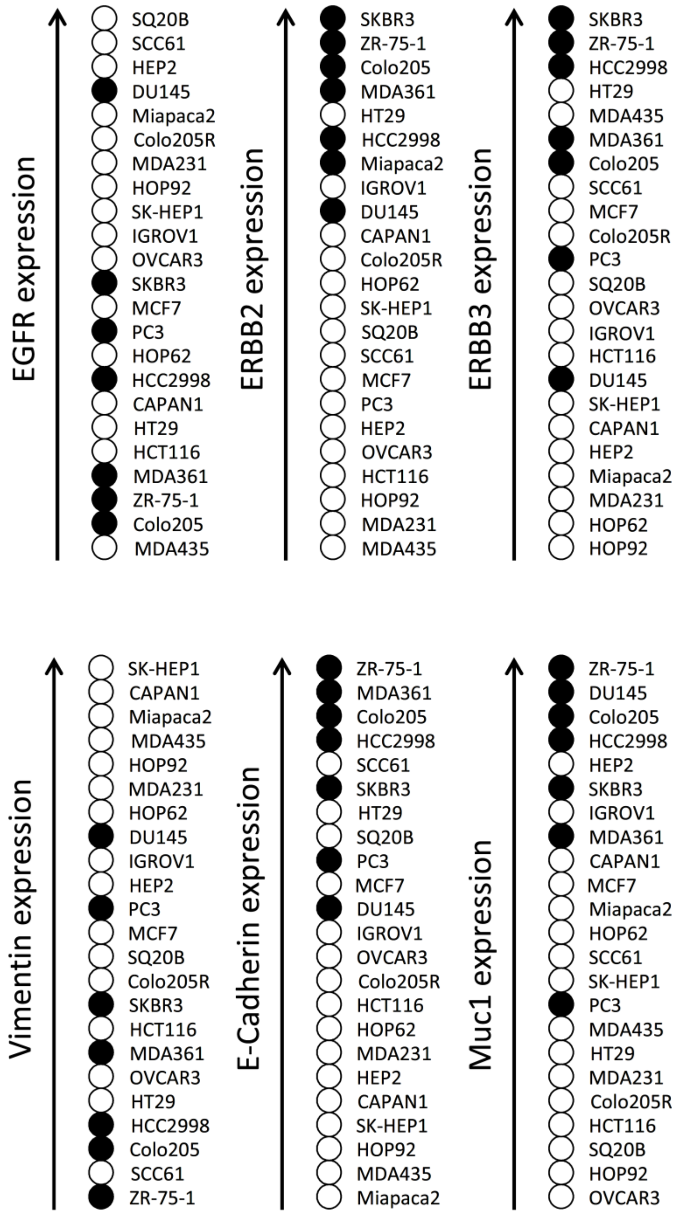

Figure S5. Relative mRNA expression of SNAIl and WISP2 in colon and breast EMT models.

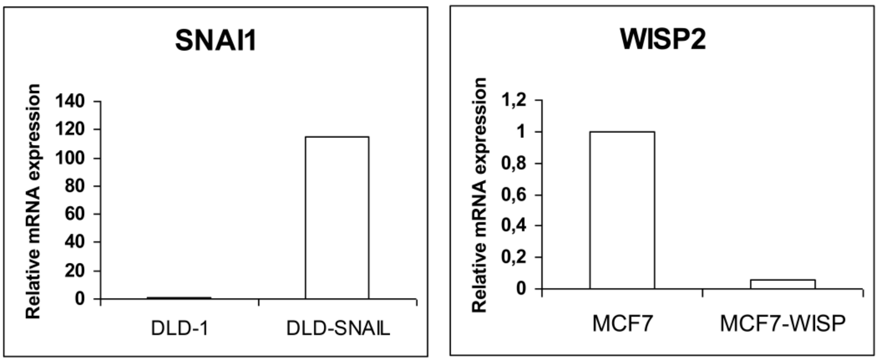

Figure S6. Protein expression levels of β-catenin, Slug, ZEB1, Claudin-1 and ZO-1, in DU145 cells exposed to 1 μM elisidepsin for 5 and 24 h, and in DU-PM cells.

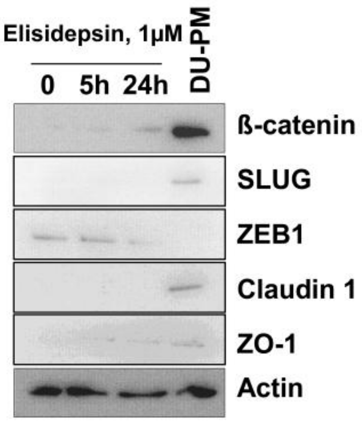

Figure S7. Elisidepsin-based combinations in DU145 colon cancer cells. The following three schedules were investigated using the Chou and Talalay method: Elisidepsin exposure for 24 h followed by 24 h exposure to lapatinib, cisplatin, oxaliplatin or gemcitabine; 24 h exposure to lapatinib, cisplatin, oxaliplatin or gemcitabine followed by 24 h exposure to elisidepsin; exposure of elisidepsin for 24 h along with chemotherapies.

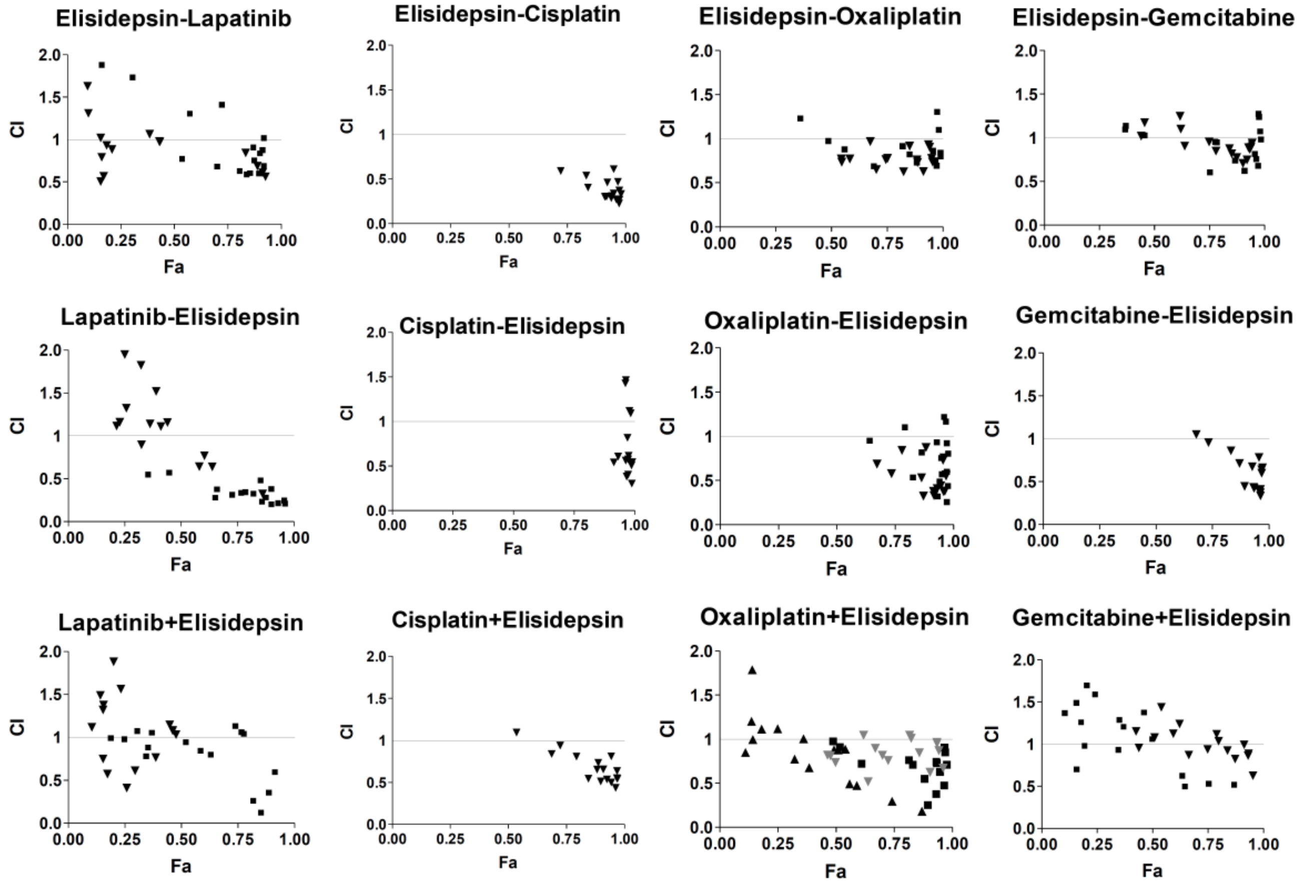

Table S1. IC50s of elisidepsin given for 24, 48 and 72 h in a panel of human cancer cell lines.

Cell line	Tumor type	IC50 (μM)		
		24 h	48 h	72 h
SKBR3	Breast	0.43 ± 0.09	0.54 ± 0.11	0.50 ± 0.1
MCF7	Breast	3.51 ± 0.70	9.86 ± 1.99	8.00 ± 2.7
Colo205	Colon	0.53 ± 0.11	1.17 ± 0.23	0.75 ± 0.2
HCC2998	Colon	0.92 ± 0.18	1.14 ± 0.23	1.20 ± 0.4
HT29	Colon	9.60 ± 1.90	10.30 ± 2.10	3.70 ± 0.8
Colo205R	Colon	8.40 ± 1.68	10.98 ± 2.20	6.10 ± 2.1
HCT116	Colon	7.87 ± 1.57	5.82 ± 1.16	7.20 ± 2.2
HEP2	Head and Neck	3.20 ± 0.64	6.80 ± 1.36	4.30 ± 1.2
SCC61	Head and Neck	7.25 ± 1.45	6.20 ± 1.24	5.60 ± 1.8
SK-HEP1	Hepatocarcinoma	6.64 ± 1.33	8.89 ± 1.78	6.00 ± 1.9
HOP62	Lung	6.02 ± 1.20	10.14 ± 2.03	6.30 ± 1.9
HOP92	Lung	7.16 ± 1.43	10.30 ± 2.06	8.00 ± 2.9
MDA-MB-435	Melanoma	4.42 ± 0.88	4.18 ± 0.84	4.40 ± 0.9
IGROV1	Ovarian	1.59 ± 0.32	5.48 ± 1.10	4.20 ± 0.8
OVCAR3	Ovarian	8.40 ± 1.68	7.31 ± 1.46	7.30 ± 2.2
CAPAN1	Pancreas	10.07 ± 2.01	12.86 ± 2.57	5.00 ± 1.6
MiaPaCa2	Pancreas	14.94 ± 2.99	11.91 ± 2.38	8.80 ± 3.1
DU145	Prostate	4.37 ± 0.87	2.72 ± 0.54	1.26 ± 0.4
PC3	Prostate	0.81 ± 0.16	1.34 ± 0.27	1.80 ± 0.4

Table S2. Genes analyzed as potential biomarkers of elisidepsin sensitivity.

ABCB1	FGFR1	MUC1	SNAIL
ACTA2	FGFR2	NRP1	TCF3
BCL2	FRAP1	NRP2	TGFB1
CDC25B	GAL S1	PAI1	TGFB2
CDH1	GAL S3	PAR1	TGFB3
CDH2	GAL S8	PDGFRA	TNF
CDKN1A	GATA3	PDGFRB	TWIST
CLDN1	HGF	PIK3CA	VEGFA
CLDN4	HIF1A	PROK1	VEGFB
COX2	HMGA2	PROM1	VEGFC
CSF1R	HMOX1	PTEN	VEGFD
CXCL12	IGF1R	PUMA	VEGFR1
CXCR4	IRS1	RET	VEGFR2
EDN1	KI67	RPS6KB1	VEGFR3
ELOVL1	KIT	SEMA3A	VIM
ERBB1	KITLG	SEMA3B	ZEB1
ERBB2	KRT18	SEMA3F	
ERBB3	KRT8	SIP1	
ERBB4	MET	SLUG	

Table S3. Relative mRNA expression of a selection of genes.

Cell line	Elisidepsin IC50s	Relative mRNA expression						
		ERBB1	ERBB2	ERBB3	ERBB4	VIM	CDH1	MUC1
**ZR-75-1**	0.4	126	1629	36015	4756	3	403793	35206
**SKBR3**	0.5	402	9899	39909	967	27	96465	10297
**Colo205**	0.75	51	562	13658	0	7	131877	18182
**HCC2998**	1.2	269	307	26295	4	7	111621	18111
**MDA-MB-361**	1.25	130	554	18924	0	20	132570	6476
**DU145**	1.26	872	264	2089	0	16221	44547	22482
**PC3**	1.8	338	139	4091	5	777	69376	913
**SQ20B**	3.5	9886	186	2957	0	31	79121	202
**HT29**	3.7	186	356	25221	0	10	94122	633
**IGROV1**	4.2	594	284	2497	2	10579	43500	6913
**HEP2**	4.3	1399	136	958	38	10230	24	15637
**MDA-MB-435**	4.4	13	60	21200	30	52306	3	687
**MDA-MB-231**	4.7	676	72	370	0	34558	104	392
**CAPAN1**	5	203	222	1128	0	58507	17	3508
**SCC61**	5.6	3668	165	4915	0	7	99905	1628
**SK-HEP1**	6	623	202	1570	0	62734	8	931
**Colo205-R**	6.1	743	218	4106	2	30	23794	386
**HOP62**	6.3	336	208	144	2	28165	310	2419
**HCT116**	7.2	165	109	2335	2	24	10643	245
**OVCAR3**	7.3	411	129	2660	16	15	29428	127
**HOP92**	8	669	103	73	3	49633	5	194
**MCF7**	8	379	146	4182	27	32	53379	3439
**MiaPaCa2**	8.8	789	294	749	0	56665	2	3425

© 2013 by the authors; licensee MDPI, Basel, Switzerland. This article is an open access article distributed under the terms and conditions of the Creative Commons Attribution license (http://creativecommons.org/licenses/by/3.0/).
